# COVID-19 Infection Control Measures in Long-Term Care Facility, Pennsylvania, USA

**DOI:** 10.3201/eid2702.204265

**Published:** 2021-02

**Authors:** Scott T. Shimotsu, Ariel R.L. Johnson, Ethan M. Berke, Daniel O. Griffin

**Affiliations:** UnitedHealth Group, Minnetonka, Minnesota, USA (S.T. Shimotsu, A.R.L. Johnson, E.M. Berke, D.O. Griffin);; Columbia University College of Physicians and Surgeons, New York, New York, USA (D.O. Griffin);; ProHealth Care Optum, New York (D.O. Griffin)

**Keywords:** COVID-19, SARS-CoV-2, severe acute respiratory syndrome coronavirus 2, viruses, respiratory infections, zoonoses, coronavirus disease, testing, long-term care facilities, infection control, Pennsylvania, United States

## Abstract

Residents of long-term care facilities are at risk for coronavirus disease. We report a surveillance exercise at such a facility in Pennsylvania, USA. After introduction of a testing strategy and other measures, this facility had a 17-fold lower coronavirus disease case rate than neighboring facilities.

The coronavirus disease (COVID-19) pandemic created an urgency to accelerate data collection to better understand the outbreak in vulnerable populations and identify best strategies for containment (*1*). Data suggested that older adults living in long-term care facilities (LTCFs) were at high risk for infection (*2**,**3*). Guidance issued by the Centers for Disease Control and Prevention outlined the importance of restricting visitation, canceling group activities, and implementing symptom screening for residents and healthcare workers (HCWs). Mitigation was put in place to limit visitors to these facilities; however, residents rely on staff, who may be exposed to severe acute respiratory syndrome coronavirus 2 (SARS-CoV-2) outside the facility. As was seen in Seattle, Washington, USA, during early 2020 (*4*), once SARS-CoV-2 is introduced into a LTCF, infection and death can be common. We hypothesized that high-risk persons in group living situations would benefit from regular, proactive monitoring for COVID-19 to prevent infection and the high transmission rates that occur in LTCF (*5*).

In this surveillance exercise performed at Twin Pines, an LTCF in Chester County, Pennsylvania, USA, we selected participants based on their association with this LTCF through employment, frequent visits (e.g., deliveries, essential care), or residence. Although we were solely observing the impact of facility-wide quality improvement, we obtained approval from the UnitedHealth Group Research and Development Institutional Review Board (Minnetonka, Minnesota, USA) and proceeded with its oversight. All persons involved in daily activities of the LTCF, including residents, employees, and visitors, were asked to participate. They completed daily symptom surveys and provided samples by nasal swab. If a participant had trouble with the self-administered sampling, a healthcare provider assisted by overseeing or performing the process. Healthcare providers collected nasal swab tests from residents twice per week and staff daily for 10 weeks (June 23–October 1, 2020). All symptom surveys and tests were conducted at the LTCF.

In addition to all 92 of the staff (nurses, therapists, and other personnel), 9 frequent visitors completed a survey and test every time they entered the facility during the surveillance period. Delivery persons who did not enter the building were not required to participate. The use of personal protective equipment (PPE) was required for all staff and visitors; PPE consisted of masking at all times while in the facility and wearing N95 masks in isolation and quarantine areas. Strict hygiene practices for the staff and twice-daily cleaning were enforced. Only full-time staff worked during this period; no per-diem staff were engaged. New residents were admitted to the facility during the observation, but they were required to quarantine for 14 days or until they had 2 negative tests. Family visits and group activities were not allowed.

During this surveillance period, a total of 5,625 nasal swabs were evaluated. We processed swab test specimens by reverse transcription PCR using a SARS-CoV-2 nucleic acid amplification test platform (LabCorp, https://www.labcorp.com). Results were provided to participants; typical turnaround time was 3 days. Two of 111 residents who tested positive had confirmed positive SARS-CoV-2 tests (results available in 1 day for the first infected resident and 7 days for the second; the delay for the second patient resulted from increased testing and limited capacity). The 2 patients were isolated for 10 days, after which they were retested until they tested negative. Staff who tested positive waited 10 days from their initial positive test and were required to have 2 negative tests before returning to work. Frequent testing and symptom surveys enabled the detection of 1 infected staff member early enough to prevent spread within the facility. Based on data obtained September 28–October 9, 2020, this LTCF’s case number was 17 times lower than that of neighboring facilities when adjusted for the facility census. 

Although our findings were encouraging, several aspects of our study need confirmation in future studies. The introduction of testing, questionnaires, and infection control measures may not fully explain the low prevalence of SARS-CoV-2 infection. We do not have a clear explanation for how the 2 residents became infected after the introduction of these measures; we were unable to determine whether surveys were useful tools. It is possible that routine testing discouraged persons with symptoms from visiting. We observed a very low rate of positive tests in the LTCF staff; only 1 staff member tested positive. Potential explanations for this low rate could be that testing had an impact on behavior, symptom screening kept ill staff home, or the virus was less prevalent in the community surrounding the LTCF. Although symptom surveys were used and absentee rates were normal, staff did not report symptoms as a reason for missed work. Despite these limitations, this study suggests that a proper testing strategy coupled with other measures may result in protection of vulnerable populations.
